# A phage-based assay for the rapid, quantitative, and single CFU visualization of *E. coli* (ECOR #13) in drinking water

**DOI:** 10.1038/s41598-018-33097-4

**Published:** 2018-10-02

**Authors:** Troy C. Hinkley, Sangita Singh, Spencer Garing, Anne-Laure M. Le Ny, Kevin P. Nichols, Joseph E. Peters, Joey N. Talbert, Sam R. Nugen

**Affiliations:** 1 Department of Food Science, Cornell University, Ithaca, NY 14853 United States; 2 Department of Food Science and Human Nutrition, Iowa State University, Ames, IA 50011 United States; 3 Intellectual Ventures Laboratory/Global Good, Bellevue, WA 98007 United States; 4 Department of Microbiology, Cornell University, Ithaca, NY 14853 United States

## Abstract

Drinking water standards in the United States mandate a zero tolerance of generic *E. coli* in 100 mL of water. The presence of *E. coli* in drinking water indicates that favorable environmental conditions exist that could have resulted in pathogen contamination. Therefore, the rapid and specific enumeration of *E. coli* in contaminated drinking water is critical to mitigate significant risks to public health. To meet this challenge, we developed a bacteriophage-based membrane filtration assay that employs novel fusion reporter enzymes to fully quantify *E. coli* in less than half the time required for traditional enrichment assays. A luciferase and an alkaline phosphatase, both specifically engineered for increased enzymatic activity, were selected as reporter probes due to their strong signal, small size, and low background. The genes for the reporter enzymes were fused to genes for carbohydrate binding modules specific to cellulose. These constructs were then inserted into the *E. coli*-specific phage T7 which were used to infect *E. coli* trapped on a cellulose filter. During the infection, the reporters were expressed and released from the bacterial cells following the lytic infection cycle. The binding modules facilitated the immobilization of the reporter probes on the cellulose filter in proximity to the lysed cells. Following substrate addition, the location and quantification of *E. coli* cells could then be determined visually or using bioluminescence imaging for the alkaline phosphatase and luciferase reporters, respectively. As a result, a detection assay capable of quantitatively detecting *E. coli* in drinking water with similar results to established methods, but less than half the assay time was developed.

## Introduction

Clean drinking water has been declared a fundamental human right, yet millions still lack access to consistently clean sources of potable water^1^. Pathogenic *Escherichia coli* (*E. coli*), a common drinking water contaminant, is a major cause of morbidity and mortality worldwide. While the WHO estimates approximately 63,000 annual deaths are due to *E. coli* infections, the added consequence of 5 million years of life lost (YLLS) and 5 million disability adjusted life years (DALYS) further compounds the suffering caused by this pathogenic contaminant^2^. In addition to GI tract infections, *E. coli* is responsible for 8.9% of sepsis cases, 29% of early onset neonatal sepsis cases and the majority of urinary tract infections^3,4^. Generic *E. coli* species which consist of both pathogens and non-pathogens, are ubiquitous in mammalian feces^5–9^. Because these organisms are naturally found in the feces of mammals in high concentrations, their presence is a biological indicator of fecal contamination and therefore possible pathogens in drinking water^10^.

The United States EPA and FDA have set a limit of zero CFU generic *E. coli* in 100 mL for drinking water and postharvest produce rinse water, respectively. Untreated agricultural water, such as that used for irrigation, has a maximum geometric mean (GM) of 126 CFU or less with a statistical threshold value (STV) of 410 or less of generic *E. coli* in a 100 mL sample^11^. These same requirements are used by the EPA for untreated recreational water. EPA Method 1603 is an approved drinking water assay that quantifies generic *E. coli* with an assay time of 24 hours. This drinking water assay contains a membrane (0.45 µm) filtration step to remove bacteria from the water sample prior to enrichment on selective and differential media. The bacteria CFUs are quantified directly on the filter following a lengthy enrichment^12^.

An alternative assay used to detect generic *E. coli* in agricultural and produce rinse water is the most probable number (MPN) method. The MPN is determined by serial diluting the water sample in triplicate or pentaplicate followed by incubation in selective growth media. The tubes are then assessed for bacterial growth and the highest dilution numbers for each replicate are used to statistically estimate the MPN of the original sample.

While both EPA 1604 and MPN can detect a single *E. coli* CFU in 100 mL of water, the prolonged incubation periods necessary for visual identification make them less practical for time sensitive applications. For example, the days required to receive results for drinking water or recreational water may be too long to prevent individuals from becoming infected. Similarly, the results for *E. coli* counts in produce rinse water may not be available until after the produce has been sold and consumed. Due to rapid spoilage, many types of produce may be sold and consumed before microbial results from traditional methods are available. Therefore, there is a significant need to rapidly detect *E. coli* in water samples while maintaining high sensitivity and quantification.

While the identification of indicators and/or pathogens often involves culturing of serological, food, or environmental samples, new technologies aim to significantly decrease assay times. Although some advanced technologies (e.g. optical nanostructures, surface enhanced Raman spectroscopy, flow cytometry, etc.) have shown promise as sensitive detection methods, they typically process only small, relatively clean samples. The true bottleneck to rapid detection methods remains the separation of a target analyte from a large complex matrix.

Currently, a pragmatic system that integrates sample preparation and rapid pathogen detection from large volume samples remains elusive. While the vast majority of research focuses on improving sensitivity of pathogen detection rather than separation/concentration, an ideal detection platform would incorporate all these components into one rapid, sensitive and specific detection assay. Additionally, while some regulations may be satisfied with binary presence/absence results, others, such as those for irrigation water and untreated recreational water require quantitative results. Therefore, there is a need to develop a rapid detection assay with the ability to detect 1 CFU/100 mL of *viable* target bacteria, while also providing fully quantitative results. These attributes mimic those of the current EPA 1603 method which is widely used for the testing of generic *E. coli* from water samples.

A new generation of rapid tests to detect bacteria utilize engineered bacteriophages^13,14^. Bacteriophage (phages) are viruses that infect bacteria in a strain-specific manner. Phages exhibit specific host ranges due to the complex interactions involved in phage attachment to the bacterial cell surface. Following adsorption, the phage injects its genome into the bacterial host and initiates infection. Then bacterial DNA transcription and translation systems begin the process of replicating many more infectious phage particles along with the lytic enzymes responsible for eventual bacterial lysis. These viral predators contain robust biorecognition elements commonly employed in sensitive bacterial detection assays^13–22^. We took advantage of a naturally evolved viral infection process that dramatically alters bacterial protein expression to overexpress engineered reporter enzymes.

While, previous studies have reported the integration of phage-based testing and filtration for water testing, the migration of reporter probes prohibited full quantification and single CFU visualization^23^. We hypothesized that the fusion of an affinity binding module to the reporter probe could effectively immobilize the probe in vicinity to the lysed bacterial host, allowing improved quantification. Therefore, a phage-based detection assay for generic *E. coli*, modeled after the widely used membrane filtration assay, EPA Method 1603, for drinking water was developed. First, highly active reporter enzymes was genetically fused to a carbohydrate binding module (CBM) with specificity for cellulose. The reporter enzymes were then genetically engineered into a phage specific to *E. coli*. Following infection, the bacteria trapped on a cellulose filter lysed releasing the reporter probes, which then immobilized locally. The addition of bioluminescent or colorimetric substrates allowed a visual quantification of the host *E. coli* colonies on the filter. *Here we integrate several technologies to construct a phage-based detection platform that is quantitative and can allow the visualization of individual CFU’s from a 100 mL water sample*. The power of our approach is that it integrates a custom phage that produces a highly active fusion enzyme which can be immobilized to allow improved quantification.

## Results

### Phage Construction, Isolation & Characterization

The *alp, alp::cbm* and *luc, nluc::cbm* reporter genes were successfully inserted into coliphage T7 to generate the reporter phages NRGp1 (MH651795), NRGp2 (MH651796), NRGp3 (MH651797), & NRGp4 (MH651798), respectively. Initial screening was performed using PCR and Sanger sequencing was used for validation. Full genome sequencing revealed no significant mutations in the remainder of the genome. No significant differences in plaque morphology, burst size and/or lysis times were observed between the recombinant and wild type phages (data not shown).

### Reporter probe characterization

NanoLuc, a luciferase engineered from deep sea shrimp, was selected as a luminescent reporter due to its strong signal, small size and low background^24^. NanoLuc is an ideal reporter enzyme candidate for this work as it has previously been employed in phage-based detection assays^13^ and also as a genetic fusion partner^25^. The alkaline phosphatase double mutant was selected as a colorimetric reporter due to its high enzymatic activity, low background and the wide variety of substrates available^26^.

We used the lytic phage T7, originally designed as a phage display platform (T7 Select), which contains a multiple cloning site directly downstream of the capsid gene^27,28^. The expression cassette contained a stop codon upstream of the reporter in order to highly express the standalone reporter enzyme as opposed to the capsid fusion commonly employed in phage display. This insertion location has previously been used in both phage display^29^ and reporter phage applications^15,30^. Furthermore, the strong phi10 T7 promoter^31^ and a custom ribosome binding site were used to promote enzyme expression^32^.

**Figure 1:**
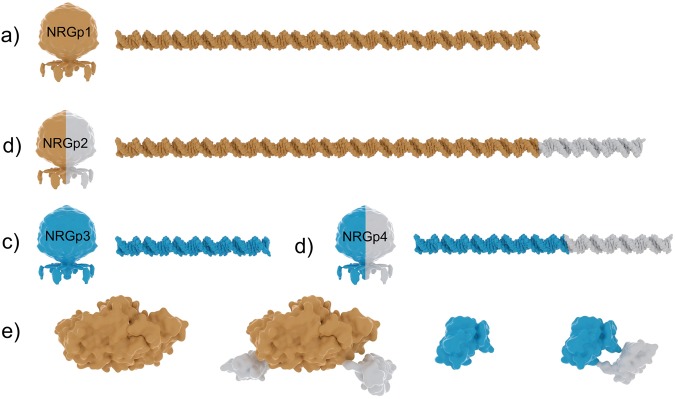
Reporter enzyme fusion design for the engineered bacteriophages. Reporter genes were synthesized within expression constructs as described previously. The inserted nucleic acid lengths for (**a**) alkaline phosphatase (**b**) alkaline phosphatase + CBM (**c**) NanoLuc and (**d**) NanoLuc + CBM are shown in relative scale. The resulting proteins (**e**) are represented in scale using pdb files 5ibo, 1kh7, 1exg for NanoLuc, Alkaline Phosphatase and CBM2a, respectively.

The enzymatic activity of the two reporters used, alkaline phosphatase and NanoLuc luciferase, have been engineered to be over two orders of magnitude greater than their respective counterparts^24,26^. In this work, these two highly active enzymes were functionalized with an affinity binding motif through the genetic fusion of a carbohydrate binding module (CBM) from *Cellulomonas fimi* with specificity to cellulose (CBM2a). As seen in Fig. 1, four total recombinant phages were generated (two of which carried celulose binding functionality) and were used in detection assays.

**Figure 2:**
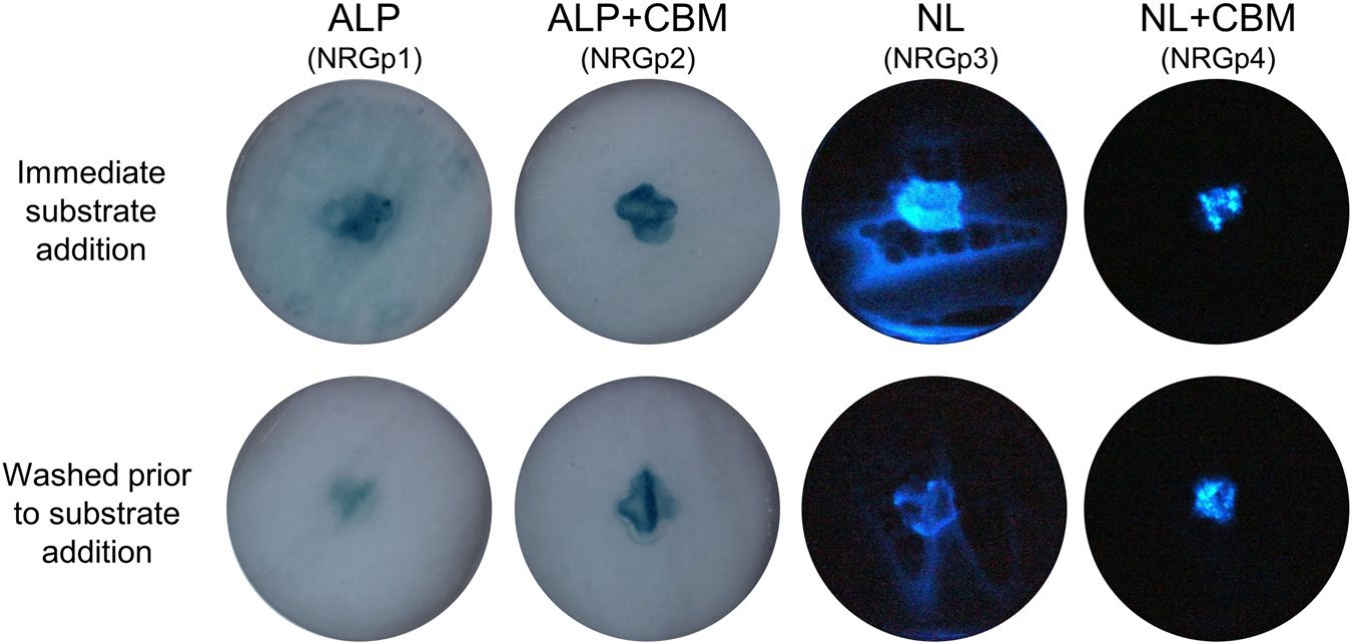
Reporter probes fused with a carbohydrate binding module significantly limits diffusion across. The reporter probes were spotted (1 mL) onto the center of the filter until the entire filter was saturated by diffusion. The effect of the carbohydrate binding module (CBM) fusion to the reporter enzymes was visualized by the degree of diffusion from the center of the filter (top row). The binding strength was further evaluated by washing the filter with PBS before substrate addition (bottom row).

To evaluate the binding affinity of the CBM fusions, phage lysates containing the respective reporter enzyme were slowly spotted onto the center of a cellulose filter allowing for passive diffusion to completely saturate the membrane. Following drying in ambient air, the respective substrates were applied and images of the filters were captured. As seen in Fig. 2, T7 with a NanoLuc + CBM gene (NRGp4) and T7 with an alkaline phosphatase + CBM gene (NRGp2) displayed limited diffusion and a stronger, concentrated signal whereas T7 with a NanoLuc gene (NRGp3) and T7 with an alkaline phosphatase gene (NRGp1) both exhibit significant diffusion and signal dilution. Similar relative binding affinities were displayed when the filters were washed with a biological buffer prior to substrate addition. To a lesser extent, the same phenomenon was observed when the respective phages were used in the detection assay.

### Detection of *E. coli* colony forming units

#### Determination of assay time

Detection assays employing membrane filtration require pre-enrichment to allow the bacteria on the filter to grow to a sufficient density cells at which a detectable signal can be generated. The amount of pre-enrichment needed for a bacterial colony to grow to a detectable size depends on several factors (generation time, growth conditions, reporter enzyme kinetics, etc.) and was investigated for the proposed assay. While the first visible indication of colony formation occurred well before 8 hours of pre-enrichment for ECOR #13, 8 hours was selected as the ideal pre-enrichment time to minimize false negative results by ensuring that smaller colonies grew large enough to produce a measurable signal. With only 90 minutes required for phage infection and 15 minutes for substrate addition and imaging, our phage based detection assay provided quantifiable results in less than half the time of traditional detection assays.

### Visualization of colonies

**Figure 3:**
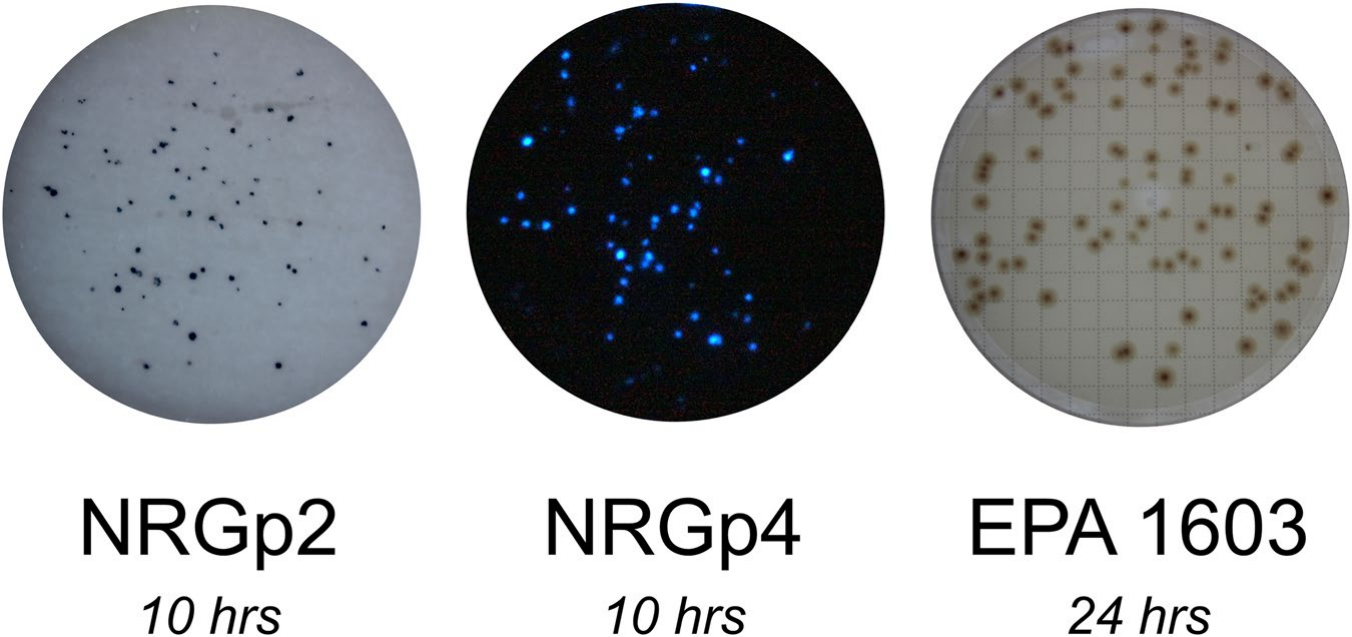
Visual comparison of the EPA method 1603 with the phage-based methods. While the EPA method requires a significantly longer time for results, the use of phage-based reporters allows a more rapid determination. The times listed are the total assay times including incubation. All filters diameters are 47 mm.

The NanoLuc enzyme, when complexed with its substrate NanoGlo, exhibits a blue luminescent signal with a peak emission at 460 nm. To mitigate signal decay, a camera was placed in close proximety to the cellulose filters. Although alkaline phosphatase has a wide range of available substrates, the colorimetric substrate 5-bromo-chloro-3-indolyl-phosphate *p*-toluidine salt (BCIP) was used to visualize bacterial colonies infected with either NRGp1 or NRGp2. The insoluble blue precipitate which formed as a reaction product between alkaline phosphatase and BCIP was easily visualized by the naked eye, with no requirements for specialized equipment (Fig. 3).

### Phage Based Detection vs. EPA Method 1603

EPA Method 1603 requires pre-enrichment on selective and differential media (modified mTEC) to enumerate total coliforms for water quality testing. Drinking water samples were spiked with varying concentrations of *E. coli* (ECOR #13) and evaluated using EPA Method 1603, the NRGp4 phage method, and the NRGp2 phage method.

The phage based methods used 8 hours of pre-enrichment, followed by a 90-minute phage infection period. From initial filtration to final results, the total assay time for the phage based methods was approximately 10 hours, whereas the EPA method requires 24 hours before accurate counts could be observed. Images of representative filters are shown in Fig. 3.

**Figure 4:**
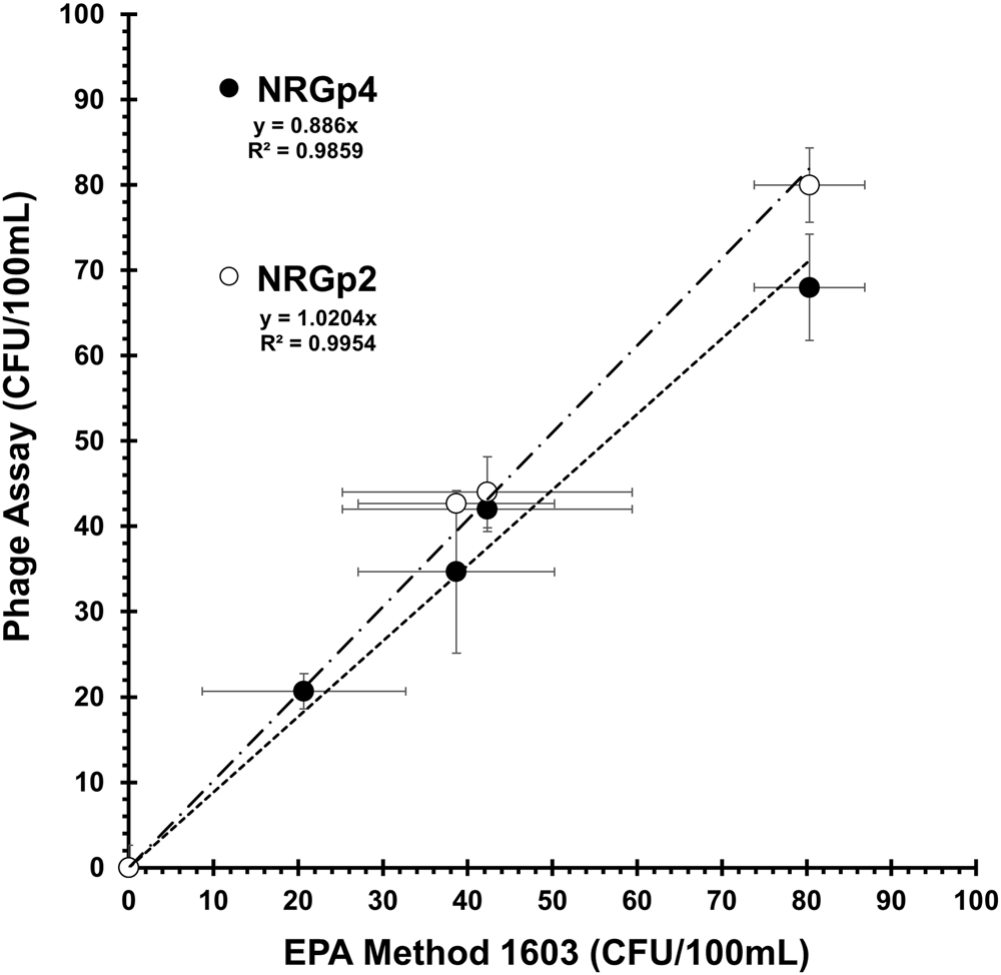
Performance comparison of the phage-based methods against the approved EPA Method 1603. Dilutions of *E. coli* in 100 mL of water were tested in triplicate using all three methods. Both NRGp2 (ALP+CBM) and NRGp4 (NanoLuc+CBM) methods showed similar CFU counts over the different concentrations. Error bars represent the standard deviation of three replicates.

Each dilution of *E. coli* was run in triplicate for each of the three methods. A negative control containing no inoculated *E. coli* was also for all methods. The CFU counts for the methods were compared to determine agreement. For each dilution, EPA Method 1603 was compared to the NRGp4 method resulting in a linear relationship with a slope of 0.89 (R^2^ ≈ 0.99). Similarly, EPA Method 1603 was compared to the NRGp2 method resulting in a linear relationship with a slope of 1.02 (R^2^ ≈ 1.00). As seen in Fig. 4, the variations observed between the phage based methods falls within the variation of the EPA test. Two-factor ANOVA with replication was used to determine the difference between EPA Method 1603 and the two phage-based methods at the several bacteria concentrations reported. The results indicate there was no significant difference at the p < 0.01 level, between EPA Method 1603 and the NRGp2 method [F(1,3) = 1.105, *p* = 0.306] and EPA Method 1603 and the NRGp4 method [F(1,3) = 1.667, *p* = 0.689]. We can conclude that both the phage-based tests fall within the natural variation of the accepted EPA Method 1603 at low bacterial concentrations, and therefore provide comparable results in less than half the time.

## Discussion

Detection of bacterial pathogens or their indicators in larger samples of water is critical to ensuring safe drinking water in developing countries. Given the zero tolerance regulatory requirements for drinking water of 0 CFU/100 mL, sample sizes below 100 mL risk the possibility of false negative results. Additionally, concentration methods which result in low capture efficiencies could also contribute to false negatives. We have developed a phage based detection assay that utilizes the cellulosic filter as a functional surface with a high capture efficiency. We have also demonstrated the effectiveness of the method to deliver quantitative results which are statistically similar to the established method within the required range. Two phage-based filtration assays were compared to the current 24-hour EPA method and yielded similar results in only 10 hours. Given that phages exist for almost all bacteria, this method can be modified for other pathogens and indicators in other liquid samples such as urine, beverages, and environmental waters.

The use of reporter probes with fusion tags, which enable them to bind the filter, allowed the labeling of the individual CFU locations, and prevented the bleeding and overlapping of the enzyme product. The discrete localization of the enzyme product in proximity to the lysed bacterial cells allowed the counting of the individual CFUs and thus enabling quantification. As with all methods, higher bacterial concentrations resulted in overlapping colonies that leads to natural variations in counts. While the 47 mm filters used in the assay are recommended for < 100 CFU in order to reduce the frequency of overlapping colonies, larger filters could be used to allow for a larger dynamic range.

Given the practical limitations of isolating a single viable CFU for the validation of single CFU detection ability of an assay, we performed a direct comparison between our proposed methods and the established EPA method using the same inoculated water samples. From these results, similar variability within all assay formats was observed.

By using the engineered phages and reporter probes with cellulose-affinity, we have incorporated the filter material into the signal generation and readout. This resulted in a lab-on-a-filter that could be used to identify discrete CFUs from large samples in a significantly shorter time than the current standard. As will all bacterial detection assays, a high degree of specificity is critical. In order to ensure the proper host range, phage-based assays and therapeutics commonly use cocktails of phages with differing specificities. In the future, the ability to use genetic engineering to engineer the host range of phages will have a significant impact on their utility. In the assay presented in this report, we selected a single *E. coli* isolate to demonstrate the detection platform. The isolate, ECOR #13, was isolated from a human and therefore represents an indicator of fecal contamination. While a field-ready version of this assay would most likely require a cocktail of engineered phages for the appropriate host range, we have demonstrated a proof of principle for an assay format which could enable faster field testing than those currently used in the field which require up a minimum of 24 hours to achieve quantitative results.

## Material and Methods

### Reagents and materials

The luminescent substrate, NanoGlo, was purchased from Promega (Madison, WI, USA) and prepared immediately before use according to the manufacturers specifications. All other reagents were purchased from Sigma Aldrich (St. Louis, MO, USA) unless stated otherwise. The colorimetric phosphatase substrate, 5-bromo-chloro-3-indolyl-phosphate *p*-toluidine salt (BCIP), was prepared as a stock solution (20 mg/mL) in N,N-Dimethylformamide (DMF) and stored at −20 °C. A BCIP working solution (2 mg/mL) was prepared in 1 M Diethanolamine (DEA) buffer immediately prior to use. Bioluminescent images were captured using long exposures (30 s) with a DSLR camera (Rebel T6, Canon, Melville NY, USA) in a dark box (LTE-13, Newport Corporation, Irvine, CA, USA). Nalgene™ disposable analytical test filter funnels (145-0045) for water testing were used to house the regenerated cellulose filters (Sartorius Stedim Biotech GmbH, Goettingen, Germany). All filters were 47 mm diameter with a 0.22 µm pore size.

### Bacteria, phages and culture media

*E. coli* BL21 was obtained from ATCC (Manassas, VA USA) and *E. coli* (ECOR #13), a strain isolated from a healthy human, was obtained from the Thomas S. Whittam STEC Center (East Lansing, MI, USA). Bacterial cultures were initially stored at −80 °C in 25% glycerol prior to use and were grown in Luria Bertani (LB) broth and plated on LB agar. Overnight cultures of *E. coli* were prepared in 10 mL of LB inoculated with a single bacterial colony and incubated (37 °C, 200 rpm, 18 hr). Serial dilutions were performed in sterile phosphate buffer saline (PBS). Bacteria and phages were enumerated using standard plate counts and double overlay plaque assays, respectively. The lytic coliphage T7 (T7Select 415-1) used in this work was designed as a cloning vector for routine phage display applications. The phage DNA used in this study was purified from propagations of T7Select 415-1 DNA in *E. coli* BL21, purchased from EMDMillipore (Burlington, MA, USA).

### Phage Stock Solutions

Exponentially growing host cells (200 mL) were infected with phage at an MOI of 0.1 until cellular lysis caused a significant decrease in OD_600_ (1.5–2 h). Low speed centrifugation was used to clear cellular debris (3,200 × *g*, 10 min, 4 °C) before sterile filtration (0.22 µm). Polyethylene glycol 6000 (PEG6000; 4%) and sodium chloride (NaCl; 0.4 M) were added and incubated overnight at 4 °C to precipitate phage particles. Phage were pelleted by ultracentrifugation (35,000 × *g*, 120 min, 4 °C), resuspended in phosphate buffered saline (PBS, pH 7.4), enumerated, and stored at 4 °C. All phage used in detection assays were diluted to 1 × 10^9^ PFU/mL in LB, sterile filtered (0.22 µm) and stored at 4 °C.

### Phage DNA Isolation

Phage lysates of sufficient concentration (>10^11^ PFU/mL) were used for genome extraction and purification. The phage stock solution was treated with sodium dodecyl sulfate (SDS; 2%) for 20 min. at 70 °C to disrupt the capsid release phage genomic DNA. After cooling on ice, DNA was precipitated with sodium acetate (0.3 M) and ethanol (70%). The sample was centrifuged (10,000 × *g*, 10 min, 4 °C) and the supernatant was passed through the Qiagen Genomic Tip 100/G according to the manufacturer’s recommendations.

### Reporter Enzyme Expression Constructs

A phosphatase and luciferase were chosen as reporter enzymes to transduce colorimetric and luminescent signals, respectively. Muller *et al*. performed random mutagenesis on bacterial alkaline phosphatase and demonstrated that two amino acid substitutions (D153G/D330N) increased enzymatic activity by more than two orders of magnitude^26^. Similarly, Hall *et al*. engineered a bioluminescent enzyme and substrate system (NanoLuc & NanoGlo, respectively) capable of generating a much stronger signal compared to other commonly employed luciferases^24^. An affinity binding motif with irreversible binding to crystalline cellulose (CBM2a) was identified in the xylanase 10 A gene from *Cellulomonas fimi*^33^, and employed as a genetic fusion to specifically immobilize the chosen reporter enzymes^34^. The CBM gene was genetically fused to the C-terminus of the reporter enzyme while a strong T7 promoter and ribosome binding site were inserted upstream to force high levels of expression. Finally, the expression cassette was flanked by regions homologous to the phage multicloning site and synthesized as a double stranded DNA fragment by IDT (Coralville, Iowa, USA).

### Construction & Isolation of Reporter Phages

Phage genomic DNA was isolated and purified from propagations of T7 Select 415-1 as described previously. Purified phage DNA was digested with *Hind*III to prepare the vector for reporter gene insertion. The reporter gene, containing homology to each vector arm, was added to the phage genomic vector at a 2:1 molar ratio and was assembled using NEBuilder® Hifi DNA Assembly Master Mix (NEB, Ipswitch, MA). Transformations were performed in electrocompetent *E. coli* DH10B (MegaX, ThermoFisher) in 1-mm cuvettes under standard conditions. Recovery was performed in SOB with shaking until visible signs of lysis occurred. Serial dilutions were performed until double overlay plaque assays revealed individual plaques. Correct clones were identified with application of enzymatic substrate and imaging as described previously. Positive plaques were further evaluated using PCR to verify insert size and full genome sequencing. The constructs for the inserted genes are shown in Fig. 1.

### Enzyme Substrates & Imaging

#### Alkaline Phosphatase

The phosphatase substrate 5-bromo-4-chloro-3-indolyl-phosphatase, 4-toluidine salt (BCIP) was dissolved in DMF (20 mg/mL) and stored at −20 °C. Immediately before use, BCIP stock was diluted tenfold in diethanolamine buffer (1 M DEA, pH 10.1) and applied directly to the filter. A short incubation (37 °C, 10 min.) generated sufficient color development for imaging. All images were captured with a DSLR camera on an LED light box (AGPTek, Brooklyn, NY, USA).

#### NanoLuc

NanoGlo buffer was prepared according to the manufacturer’s recommendations immediately before use. The filter membranes were fully saturated in substrate (~300 µL) prior to imaging. Long exposure bioluminescent images were captured with a DSLR camera in a dark box using 30 second exposure times.

### Assay procedure

**Figure 5:**
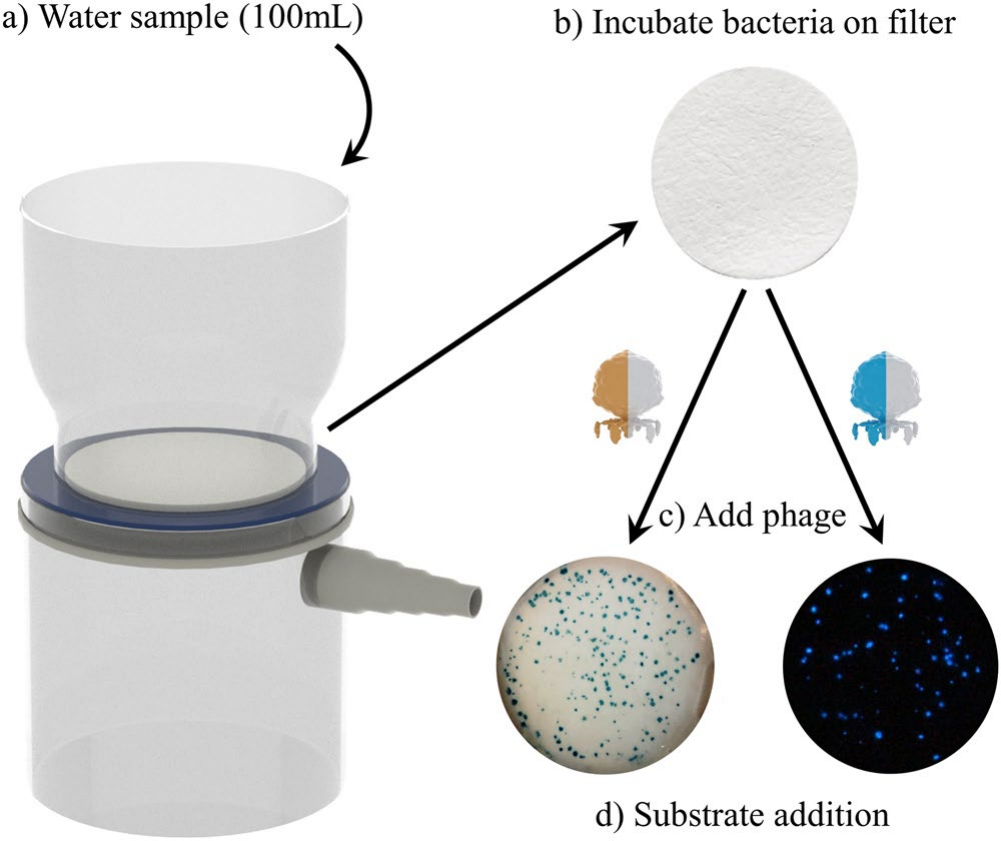
Schematic diagram of the phage-based detection assay. (**a**) The 100 mL water sample passes through the filter, retaining any bacterial contaminants. (**b**) The filter is placed on media and incubated to allow for colony formation. (**c**) Recombinant phages are added to force expression of the desired reporter. (**d**) Substrate addition provides a colorimetric signal for NRGp2 and a bioluminescent signal for NRGp4.

The phage-based procedure used the initial filtration steps of EPA method 1603 followed by phage infection and imaging (Fig. 5). Drinking water samples (100 mL) were obtained from a local municipal water source (Ithaca, New York, USA) and autoclaved. The sterile drinking water samples were inoculated with varying concentrations of stationary phase *E. coli* (ECOR #13) and filtered according to the procedure outlined in EPA Method 1603. Following filtration, the filter membrane was removed and placed onto an absorbent pad saturated with LB broth. The filters were incubated (37 °C, 8–12 hours) to allow for colony growth. Following the initial enrichment, a phage solution (2 mL, 10^9^ PFU/mL in LB) was applied to the filter and incubated (37 °C, 90 min) to initiate phage infection and reporter probe expression. After brief drying on a sterile absorbent pad, the enzymatic substrates were applied and imaged as described previously.

In order to characterize the performance of the phage-based method, the concentration of *E. coli* in the water samples was determined in parallel using the EPA method 1603^12^. Following filtration, the filter was placed onto modified mTEC agar and incubated according to the methods specifications. Colonies were counted after the required 24-hour incubation period.

### Image analysis

Images of the bioluminesnce on the capture filters were analyzed using ImageJ. The relatively low background of the bioluminescent images allowed the pixel intensities to be multiplied by three resulting in an improved visualization of the spots. The spots were counted both visually as well as using the imageJ software to allow a determination of the accuracy of the image analysis. Spot sizes and distribution were determined using ImageJ particle size distribution.

## Electronic supplementary material

Supplementary Information
