# Supplementary material for: A phage-based assay for the rapid, quantitative, and single CFU visualization of E. coli (ECOR #13) in drinking water
Source: Sci Rep. 2018 Oct 2;8:14630. doi: 10.1038/s41598-018-33097-4 (PMC6168599; doi:10.1038/s41598-018-33097-4)

A phage-based assay for the rapid, quantitative, and single CFU visualization of *E. coli* in drinking water

Troy C. Hinkley,[1] Sangita Singh,[2] Spencer Garing,[3] Anne-Laure M. Le Ny,[3] Kevin P. Nichols,[3] Joseph E. Peters,[4] Joey N. Talbert,[2] and Sam R. Nugen[1,*]

[1] *Department of Food Science, Cornell University, Ithaca, NY 14853, United States*
[2] *Department of Food Science and Human Nutrition, Iowa State University, Ames, IA 50011, United States*
[3] *Intellectual Ventures Laboratory / Global Good, Bellevue, WA 98007, United States*
[4] *Department of Microbiology, Cornell University, Ithaca, NY 14853, United States*
*Corresponding author: 241 Stocking Hall, Ithaca, NY 14853, USA P: +011 (607) 255-9185 snugen@cornell.edu

Raw Data for Figure 4:

Nluc-CBM Raw Data — Dilution Samples

	Replicates	1	2	3	4	(-)Control
1603	1	20	31	34	87	0
	2	33	33	31	80	0
	3	9	52	62	74	0
NRGp4	1	23	25	44	63	0
	2	19	35	39	66	0
	3	20	44	43	75	0
NRGp2	1		44	39	83	0
	2		46	47	78	0
	3		38	46	79	0

Averages — Dilution Samples

		1	2	3	4	Control
1603	Average	21	39	42	80	0
	SD	12	12	17	7	0
NRGp4	Average	21	35	42	68	0
	SD	2	10	3	6	0
NRGp2	Average		43	44	80	0
	SD		4	4	3	0

Method	Dilution 1	Dilution 2	Dilution 3	Dilution 4	(-)Control
1603	20	31	34	87	0
	33	33	31	80	0
	9	52	62	74	0
NRGp4	23	25	44	63	0
	19	35	39	66	0
	20	44	43	75	0

Anova: Two-Factor With Replication
α = 0.01

SUMMARY	Dilution 1	Dilution 2	Dilution 3	Dilution 4	(-)Control	Total
1603						
Count	3	3	3	3	3	15
Sum	62	116	127	241	0	546
Average	20.66667	38.66667	42.33333	80.33333	0	36.4
Variance	144.3333	134.3333	292.3333	42.33333	0	846.8286
T7NL						
Count	3	3	3	3	3	15
Sum	62	104	126	204	0	496
Average	20.66667	34.66667	42	68	0	33.06667
Variance	4.333333	90.33333	7	39	0	566.4952
Total						
Count	6	6	6	6	6	
Sum	124	220	253	445	0	
Average	20.66667	36.66667	42.16667	74.16667	0	
Variance	59.46667	94.66667	119.7667	78.16667	0	

ANOVA

ce of Varia	*SS*	*df*	*MS*	*F*	*P-value*	*F crit*
Sample	83.33333	1	83.33333	1.105217	0.305661	8.095958
Columns	18109.53	4	4527.383	60.04487	7.37E-11	4.43069
Interactior	169	4	42.25	0.560345	0.694053	4.43069
Within	1508	20	75.4			
Total	19869.87	29				

Method	Dilution 2	Dilution 3	Dilution 4	(-)Control
1603	31	34	87	0
	33	31	80	0
	52	62	74	0
NRGp2	44	39	83	0
	46	47	78	0
	38	46	79	0

Anova: Two-Factor With Replication
α = 0.01

SUMMARY	Dilution 2	Dilution 3	Dilution 4	(-)Control	Total
1603					
Count	3	3	3	3	12
Sum	116	127	241	0	484
Average	38.66667	42.33333	80.33333	0	40.33333
Variance	134.3333	292.3333	42.33333	0	967.1515
T7ALP					
Count	3	3	3	3	12
Sum	128	132	240	0	500
Average	42.66667	44	80	0	41.66667
Variance	17.33333	19	7	0	883.8788
Total					
Count	6	6	6	6	
Sum	244	259	481	0	
Average	40.66667	43.16667	80.16667	0	
Variance	65.46667	125.3667	19.76667	0	

ANOVA

Source of Variati	*SS*	*df*	*MS*	*F*	*P-value*	*F crit*
Sample	10.66667	1	10.66667	0.166558	0.688598	8.530965
Columns	19319	3	6439.667	100.5543	1.35E-10	5.292214
Interaction	17.66667	3	5.888889	0.091954	0.963409	5.292214
Within	1024.667	16	64.04167			
Total	20372	23				

Method	Dilution 2	Dilution 3	Dilution 4	(-)Control
NRGp4	25	44	63	0
	35	39	66	0
	44	43	75	0
NRGp2	44	39	83	0
	46	47	78	0
	38	46	79	0

Anova: Two-Factor With Replication
α = 0.01

SUMMARY	Dilution 2	Dilution 3	Dilution 4	(-)Control	Total
T7NL					
Count	3	3	3	3	12
Sum	104	126	204	0	434
Average	34.66667	42	68	0	36.16667
Variance	90.33333	7	39	0	667.7879
T7ALP					
Count	3	3	3	3	12
Sum	128	132	240	0	500
Average	42.66667	44	80	0	41.66667
Variance	17.33333	19	7	0	883.8788
Total					
Count	6	6	6	6	
Sum	232	258	444	0	
Average	38.66667	43	74	0	
Variance	62.26667	11.6	61.6	0	

ANOVA

Source of Varia	*SS*	*df*	*MS*	*F*	*P-value*	*F crit*
Sample	181.5	1	181.5	8.081633	0.011754	8.530965
Columns	16572.5	3	5524.167	245.974	1.36E-13	5.292214
Interactior	136.5	3	45.5	2.025974	0.150813	5.292214
Within	359.3333	16	22.45833			
Total	17249.83	23				

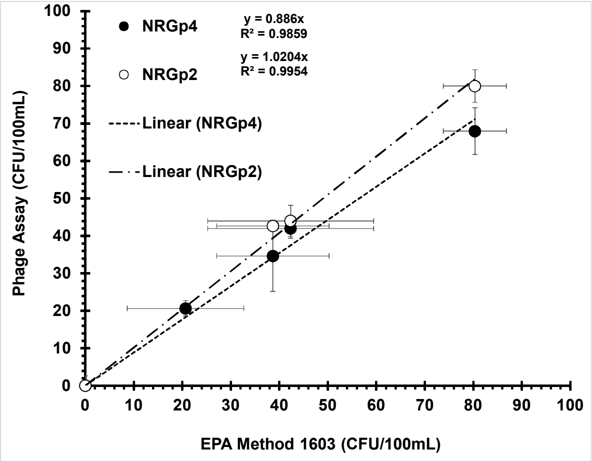

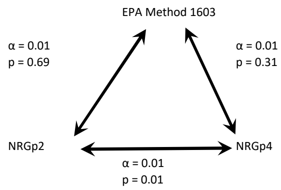

Supplement: Supplementary file 1 — Supplementary Information [file 41598_2018_33097_MOESM1_ESM.pdf]
